# A Transformer-Based Multimodal Fusion Network for Emotion Recognition Using EEG and Facial Expressions in Hearing-Impaired Subjects

**DOI:** 10.3390/s25206278

**Published:** 2025-10-10

**Authors:** Shuni Feng, Qingzhou Wu, Kailin Zhang, Yu Song

**Affiliations:** 1 Tianjin Key Laboratory of Life and Health Detection, Life and Health Intelligent Research Institute, Tianjin University of Technology, Tianjin 300384, China; 2 School of Electrical Engineering and Automation, Tianjin University of Technology, Tianjin 300384, China

**Keywords:** emotion recognition, electroencephalogram (EEG), facial expression, multimodal fusion, multi-head attention mechanism

## Abstract

Hearing-impaired people face challenges in expressing and perceiving emotions, and traditional single-modal emotion recognition methods demonstrate limited effectiveness in complex environments. To enhance recognition performance, this paper proposes a multimodal fusion neural network based on a multimodal multi-head attention fusion neural network (MMHA-FNN). This method utilizes differential entropy (DE) and bilinear interpolation features as inputs, learning the spatial–temporal characteristics of brain regions through an MBConv-based module. By incorporating the Transformer-based multi-head self-attention mechanism, we dynamically model the dependencies between EEG and facial expression features, enabling adaptive weighting and deep interaction of cross-modal characteristics. The experiment conducted a four-classification task on the MED-HI dataset (15 subjects, 300 trials). The taxonomy included happy, sad, fear, and calmness, where ‘calmness’ corresponds to a low-arousal neutral state as defined in the MED-HI protocol. Results indicate that the proposed method achieved an average accuracy of 81.14%, significantly outperforming feature concatenation (71.02%) and decision layer fusion (69.45%). This study demonstrates the complementary nature of EEG and facial expressions in emotion recognition among hearing-impaired individuals and validates the effectiveness of feature layer interaction fusion based on attention mechanisms in enhancing emotion recognition performance.

## 1. Introduction

Emotion is a complex psychological state formed in humans through external stimuli or internal drives, encompassing multiple dimensions such as subjective experience, external expression, and physiological response [1]. Emotion recognition, as a vital branch of artificial intelligence, aims to enable computers to perceive, understand, and express emotions, thereby achieving more natural human computer interaction. In recent years, emotion recognition has been widely applied in scenarios such as intelligent assistants, adaptive education, medical psychological interventions, and entertainment interactions [2]. For individuals with hearing impairments, the absence of auditory and linguistic abilities severely limits their emotional perception and expression, often leading to social communication barriers and psychological adaptation issues [3]. Therefore, developing robust emotion recognition methods not only helps improve communication and quality of life for the hearing impaired but also provides new technological support for related psychological interventions and rehabilitation training.

Most current emotion recognition research predominantly concentrates on unimodal approaches. Facial expression, as the most direct manifestation of affective states, has achieved notable advancements in classification accuracy through the application of deep learning architectures such as the Convolutional Neural Networks (CNN) and the residual networks (ResNet) [4]. However, facial expressions are significantly influenced by factors such as lighting, occlusion, and deliberate disguise, making their sole reliance for emotion recognition inherently limited. On the other hand, EEG signals directly reflect central nervous system activity, offering high physiological authenticity and objectivity. Researchers often extract emotion related information through features such as power spectra, Short Time Fourier transforms, and Differential Entropy (DE), and utilize models like Support Vector Machines (SVM), Long Short Term Memory Networks (LSTM), EEGNet, or multi-branch CNNs for classification. EEG based methods demonstrate strong performance on public datasets like DEAP and SEED [5], but signals susceptibility to artifacts and significant inter-individual variability pose challenges for practical applications.

To overcome the limitations of single-modal approaches, multimodal emotion recognition has gradually emerged as a research hotspot. By integrating complementary information from different modalities, models can achieve higher robustness and accuracy in complex environments [6]. Multimodal fusion methods primarily encompass input level fusion, feature level fusion, and decision level fusion. Input level fusion directly concatenates raw data from different modalities, which can easily lead to dimensional imbalance and noise propagation. Decision level fusion involves independent classification across modalities followed by weighting or voting, preserving modality independence but struggling to fully leverage cross-modal interaction information. Feature level fusion models analyze intermodal relationships within the feature space and are considered an effective approach for enhancing performance. However, most current methods still rely on simple concatenation or static weighting, lacking deep modeling of intermodal interactions [7]. They are particularly challenged in dynamically adjusting the importance of different modalities based on context.

In the field of emotion recognition research for individuals with hearing impairments, existing studies have demonstrated distinct brain region response characteristics during emotional processing compared to the general population [8]. For instance, their emotional functional brain regions exhibit heightened activity in the parietal and occipital lobes, with more pronounced interhemispheric differences in emotional responses [9]. This provides new theoretical grounding for multimodal approaches integrating electroencephalographic signals and facial expressions. However, most existing studies on hearing impaired individuals remain confined to unimodal approaches or lack systematic cross-modal interaction modeling. Effectively capturing the spatiotemporal dependencies of EEG signals and deeply integrating them with the overt features of facial expressions represents a critical challenge in enhancing emotion recognition performance for this population.

Based on this, this paper proposes a Multimodal Multi-Head Attention Fusion Neural Network (MMHA-FNN) tailored for individuals with hearing impairments. This method utilizes DE and bilinear interpolation to generate EEG brain topography maps. It then employs an EEG feature extraction module based on the Mobile Inverted Bottleneck Convolution (MBConv) architecture to learn the spatiotemporal characteristics of brain regions. Concurrently, a facial feature extraction module using a residual network architecture captures the deep semantic information of facial expressions. Finally, during the feature fusion stage, the Transformer multi-head attention mechanism is introduced to dynamically model the interactive relationship between EEG and facial features, enabling adaptive weighting and information enhancement across modalities. Experimental results on the Multimodal Emotion Database for Hearing-Impaired Individuals (MED-HI) demonstrate that this method achieves an outstanding performance in the four-classification task.

## 2. Related Work

Emotion recognition, as one of the core tasks in the field of affective computing, has garnered significant attention in recent years. Research on different modalities has deepened, particularly the integration of physiological signals such as EEG with non-physiological signals like facial expressions, offering new approaches to enhance the robustness and accuracy of emotion recognition.

In single-modal research, EEG serves as a crucial signal source for emotion recognition due to its ability to directly reflect neural activity in the brain. Previous studies have demonstrated that DE serves as an effective feature for characterizing emotional states. Duan et al. [10] found that DE outperforms features such as energy spectrum (ES) in emotion classification, with particularly significant recognition performance in the γ band. On the publicly available DEAP dataset, a fusion model combining 2D convolutional layers based on DE features with LSTM achieves classification accuracies of 91.9% and 92.3% on the valence and arousal dimensions, respectively [11]. On the SEED-V dataset, Yao et al. [12] combined DE with the ResNet18 network to achieve five-category emotion classification, attaining an average accuracy of 95.61%. Multimodal fusion methods have also drawn significant research attention. Wu et al. [13] conducted a comprehensive review of multimodal emotion recognition, highlighting that feature extraction and fusion strategies are crucial for enhancing system performance, while emphasizing that lightweight models represent a future challenge.

Improvements to EEG feature extraction and fusion mechanisms have also garnered significant attention. EEG FuseNet employs an unsupervised GAN-CNN-RNN hybrid architecture to achieve deep representation and fusion of EEG spatiotemporal features, demonstrating strong generalization capabilities [14]. Wu et al. [15] utilized EEG clustering coefficients and feature centrality to perform multimodal emotion recognition through DCCA. Furthermore, the introduction of Transformers in multimodal fusion has further propelled research advancements. The graph neural network framework proposed by Devarajan et al. integrates EEG, facial expressions, and other physiological signals for emotion recognition, demonstrating the advantages of graph structures in modal fusion [16]. Recent reviews also highlight that designing fusion algorithms, modal representations, and classification mechanisms remains a core challenge in current work, demanding deeper alignment and modeling of intermodal relationships [17]. Hu et al. [18] proposed STRFLNet, a spatio-temporal representation fusion learning network that integrates EEG-based features with advanced cross-modal fusion, achieving state-of-the-art accuracy on public datasets. Cai et al. [19] designed an EEG-SWTNS neural network that leverages spectral images of EEG signals, effectively capturing frequency domain patterns for emotion recognition. In addition, Pfeffer et al. explored Transformer-based models for EEG signal analysis in brain–computer interface tasks, highlighting the strong representational power of Transformer structures in biomedical signals [20].

In summary, DE features combined with deep learning networks demonstrate high efficiency in EEG emotion recognition. Cross-modal fusion, particularly when incorporating attention mechanisms, significantly improves recognition accuracy. However, these studies primarily focus on general populations and publicly available datasets. Research on multimodal emotion recognition for the hearing-impaired population remains relatively scarce, with a notable gap in methods for deep interaction fusion. Therefore, this paper proposes an MMHA-FNN tailored for the hearing-impaired population. The model utilizes DE spatiotemporal feature matrices as input, extracting temporal and spatial features through MBConv and ResNet modules, respectively. During the fusion stage, a Transformer multi-head self-attention mechanism is introduced to enable dynamic feature level interaction between EEG signals and facial expressions.

## 3. Materials and Methods

### 3.1. The Model Architecture of MMHA-FNN

**Figure 1:**
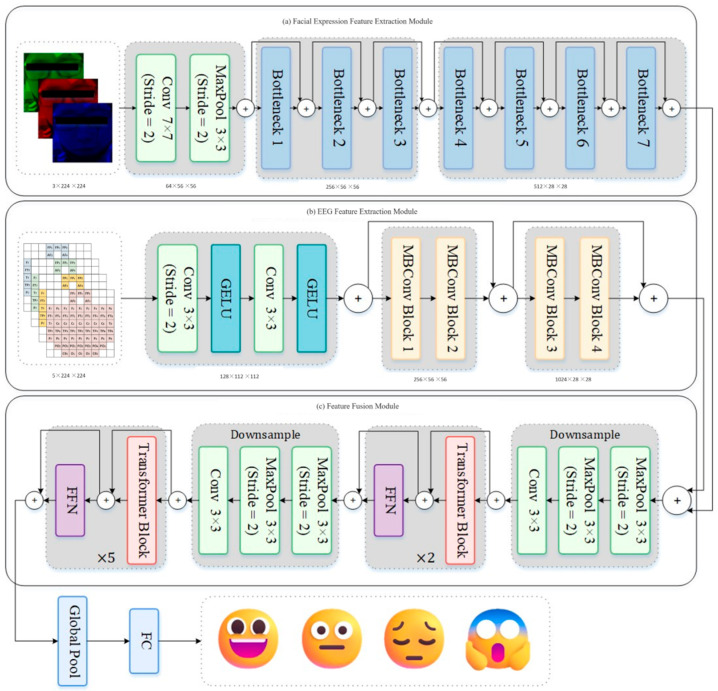
The network architecture of MMHA-FNN.

The model architecture of MMHAFNN is shown in Figure 1. This network architecture is primarily divided into three components: the facial expression feature extraction module, the EEG feature extraction module, and the feature fusion module. The Facial Expression Feature Extraction Module (FEFEM) is a residual linkage-based CNN module that learns multi-level facial expression features to represent emotion related information in subjects’ faces. Its layer-by-layer feature extraction and subsampling mechanism enables the network to progressively capture subtle changes in facial expressions—from local details to global structures. EFEM is an EEG spatio-temporal feature extraction network based on the MBConv module, which learns spatio-temporal features between electrodes by generating differential entropy brain topography representations using DE features and bilinear interpolation. Ultimately, the deep features extracted by FEFEM and EFEM will be fed into FFM for information exchange across different modalities. The fusion strategy within FFM, based on a multi-head self-attention mechanism, can capture dependencies within features and adaptively balance the importance of different modalities under multimodal input.

### 3.2. Multimodal Data Preprocessing and Feature Extraction

The MED-HI dataset consists of 300 trials from 15 hearing-impaired subjects (12 males and 3 females, aged 18–25). Emotional states (happy, sad, fear, and calmness) were elicited using 20 subtitled film clips (~200 s each), with five clips per category. EEG signals were recorded with a 64-channel Neuroscan SymAmps2 system at 1000 Hz (downsampled to 200 Hz), while facial expressions were simultaneously captured at 30 Hz. Each trial included a 5 s cue, video viewing, 45 s self-report, and a short rest, with longer breaks after every four clips. Preprocessed data were segmented into 1 s non-overlapping windows, yielding 4407 samples for emotion recognition [6].

**Figure 2:**
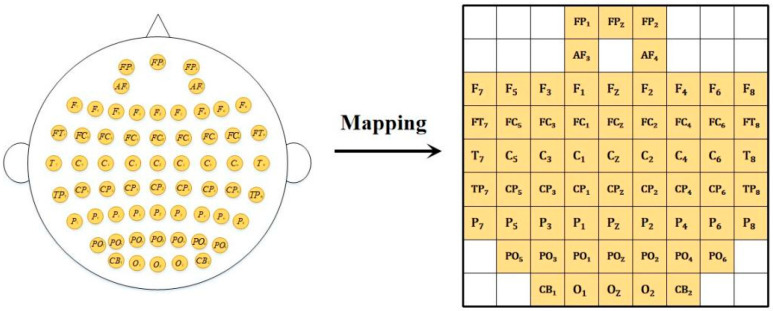
Differential entropy mapping of cerebral topography.

After preprocessing the EEG, DE features can be extracted for emotion recognition. For 1 s samples, DE features are extracted from the δ band (1–4 Hz), *θ* band (4–8 Hz), *α* band (8–12 Hz), *β* band (12–30 Hz), and *γ* band (30–50 Hz). Furthermore, to generate differential entropy brain topography maps, bilinear interpolation was applied to the DE features using the Cartesian coordinates of 62 electrodes obtained from the EEGLAB toolbox. Additionally, channels in the differential entropy brain topography correspond to distinct frequency bands, resulting in a data dimension of 4407 × 5 × 224 × 224 (number of samples × number of channels × width × height). The 3rd and 4th dimensions correspond to the interpolated EEG topography maps (224 × 224 resolution), obtained by bilinear interpolation of 62 electrodes across 5 frequency bands. Furthermore, zero padding was applied to fill the edge regions of the differential entropy brain topography. An example of the differential entropy brain topography is shown in Figure 2. The original formula for differential entropy is

(1) $$h\left(X\right)=-\int_{-\infty }^{\infty }\;f\left(x\right)\log\left(f\left(x\right)\right)dx$$

Here, *X* denotes a continuous time series, and *f*(*x*) is the probability density function of *X*. After frequency band decomposition, the EEG signal essentially follows a Gaussian distribution *N* (*μ, σ*^2^). Therefore, the calculation formula for the differential entropy feature can be expressed as

(2) $$h\left(X\right)=-\int_{-\infty }^{\infty }\frac{1}{\sqrt{2\pi \sigma^{2}}}e^{-\frac{(x-\mu {)}^{2}}{2\sigma^{2}}}\log\left(\frac{1}{\sqrt{2\pi \sigma^{2}}}e^{-\frac{(x-\mu {)}^{2}}{2\sigma^{2}}}\right)dx=\frac{1}{2}\log\left(2\pi e\sigma^{2}\right)$$

The formula for bilinear interpolation is

(3) $$f\left(x,y\right)=f\left(Q_{11}\right)\omega_{11}+f\left(Q_{12}\right)\omega_{12}+f\left(Q_{21}\right)\omega_{21}+f\left(Q_{22}\right)\omega_{22}$$

Here, $f\left(Q_{ij}\right),\;(i=1,\;2,\;j=1,\;2)$ represents the values of the four electrodes surrounding the interpolation point, while $\omega_{ij},\;(i=1,\;2,\;j=1,\;2)$ relates to the coordinate distances between the point of interest and the diagonal points.

Facial expression images with background information removed are resized to 256 × 256 pixels using center cropping. To accelerate convergence of image data during neural network training, facial expression data undergoes normalization processing:

(4) $$X_{i}\left(j,n_{f}\right)=\frac{F_{i}(j,n_{f})-\min[\;F_{i}(:,n_{f})]}{\max[\;F_{i}(:,n_{f})]-\min[\;F_{i}(:,n_{f})]}$$

Here, $X_{i}(j,n_{f})$ denotes the normalized value of the $n_{f}$th feature $F_{i}(j,n_{f})$ in the *j*-th sample of the *i*th subject, $\max[\;F_{i}(:,n_{f})]$ denotes the maximum value of the $n_{f}$th feature across all samples of the *i*-th subject, and $\min[\;F_{i}(:,n_{f})]$ denotes the minimum value of the $n_{f}$th feature across all samples of the *i*-th subject. This transforms the feature values between −1 and 1, yielding the preprocessed facial expression features (3 × 256 × 256).

### 3.3. Facial Feature Learning Based on FEFEM

**Figure 3:**
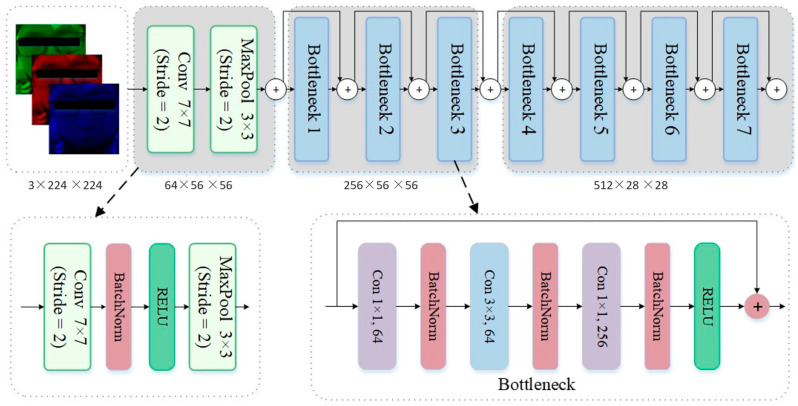
The schematic diagram of the FEFEM structure.

The FEFEM core employs Residual Network (ResNet) modules. ResNet is based on the structure of convolutional neural networks, with its core concept being the introduction of residual structures within the network. Figure 3 illustrates a schematic diagram of a residual structure in a Bottleneck layer. In traditional convolutional networks, increasing the number of convolutional layers is necessary to enhance model performance. However, excessive convolutional layers can lead to the vanishing gradient problem. The introduction of residual connections effectively resolves this issue.

In the input layer of FEFEM, the input image passes through a 7 × 7 convolutional kernel with a stride of 2 to capture low-level edge features. Combined with a BatchNorm layer that performs batch normalization on each input channel, this accelerates training convergence and enhances model stability. The ReLU layer introduces a nonlinear activation function, enabling the model to process complex input data while increasing the sparsity of network outputs to prevent overfitting. Finally, a 3 × 3 max pooling layer with a stride of 2 reduces the feature map resolution. By selecting the maximum value, it preserves key information while reducing computational complexity.

The Bottleneck block in the feature extraction layer extracts deep features through three convolutional layers (1 × 1, 3 × 3, and 1 × 1 convolutions). It reduces and restores the number of channels via 1 × 1 convolutions while processing the spatial dimension with 3 × 3 convolutions, thereby achieving a balance between computational complexity and network performance. Each Bottleneck block introduces residual connections, mitigating the vanishing gradient problem in deep networks by directly adding inputs to outputs, ensuring smoother feature propagation. Some Bottleneck blocks also incorporate downsampling operations (Stride = 2) to reduce feature map resolution and enhance feature globality. Within each Bottleneck block, the number of channels progressively expands to extract richer facial expression features.

### 3.4. EEG Spatio-Temporal Feature Extraction Based on EFEM

The preprocessed differential entropy brain topography map is input into the EFEM framework to extract EEG spatiotemporal features. The EFEM architecture integrates CNN and Squeeze-and-Excitation (SE) attention mechanism modules, employing MBConv modules and dimensionality reduction strategies for feature extraction. The module structure is illustrated in Figure 1.

At the input layer, the 5-channel differential entropy brain topography features undergo preliminary feature extraction through a convolutional layer with a 3 × 3 kernel and stride of 2. Batch normalization and nonlinear mapping are then performed via BatchNorm and GELU layers. The GELU activation function captures the complexity of input data more effectively than the traditional ReLU activation function, particularly when processing neural signals, as it better handles nonlinear information. The second 3 × 3 convolutional layer is used for local feature encoding of the EEG.

**Figure 4:**
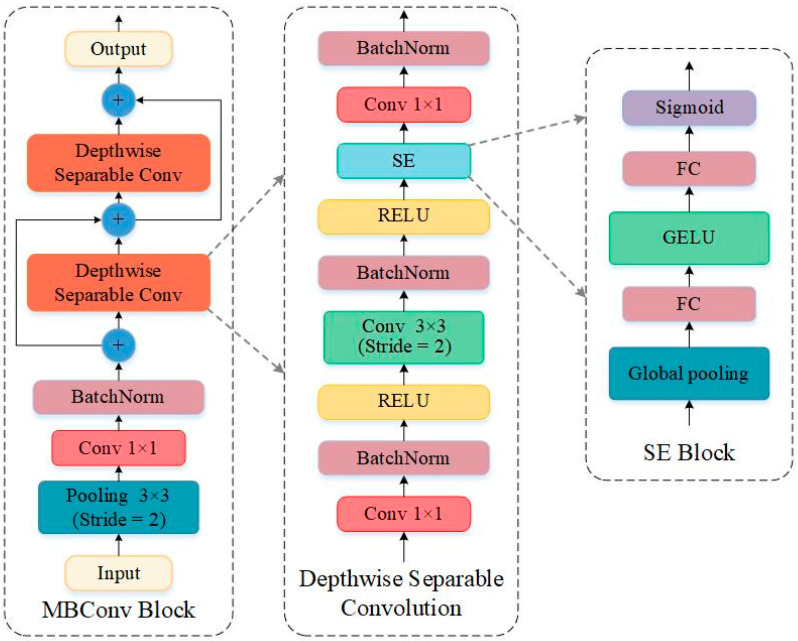
The structure of MBConv block.

At the deep feature extraction layer, the extracted differential entropy brain topography features are processed using MobileNet’s MBConv architecture to efficiently capture spatial and frequency domain characteristics. The MBConv module combines depthwise separable convolutions with an attention mechanism (SE Block) to further compress and expand features, as illustrated in Figure 4. Specifically, the input features undergo spatial downsampling via a Max Pooling layer, followed by feature dimension reduction using a 1 × 1 convolutional layer. The reduced-dimension features then pass through two Depthwise Separable Conv layers to extract EEG spatiotemporal features.

The Depthwise Separable Conv layer consists of two main components. One is the Depthwise Convolution, which performs independent convolution operations on different channels of the input data—this distinguishes it from traditional convolution. It transforms a tensor of size $h_{i\;}{\times \;w}_{i}\times d_{i}$ into one of size $h_{i\;}{\times \;w}_{i}\times d_{j}$. Traditional convolution requires parameters of size $h_{i\;}{\times \;w}_{i}\times d_{j}\times d_{i}\times k\times k$, whereas Depthwise Convolution requires only $h_{i\;}{\times \;w}_{i}\times d_{i}\times (k\times k+d_{j})$ parameters. This is because in traditional convolution operations, each convolution kernel simultaneously acts on every input channel of the input data, leading to a dramatic increase in the number of parameters and computational complexity in deep learning. In Depthwise Convolution, each channel of the input data corresponds to a dedicated convolutional kernel. This approach significantly reduces the model’s parameter count and computational load, resulting in a lighter-weight model and lower training costs. The other component is Pointwise Convolution, which combines features from different channels. It transforms information from all input channels into a new feature channel, thereby generating a higher-dimensional feature representation that encompasses all channels. This enhances the model’s expressive power.

To enhance feature expressiveness, the SE module was incorporated during the design of Depthwise Separable Convolution. This module employs global average pooling to compute global features for each channel, utilizes a linear layer to model inter-channel relationships, and performs channel reweighting after obtaining channel weights via Sigmoid activation, thereby boosting the expressiveness of important features.

### 3.5. Multimodal Feature Fusion Based on FFM

**Figure 5:**
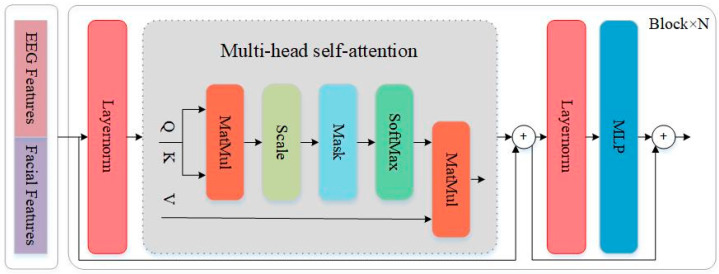
The network architecture of MMHA-FNN.

The core of FFM is the Transformer module. After multimodal deep features are concatenated, they are fed into the FFM network for feature fusion and classification. As shown in Figure 5, Layer Normalization (LN), Multi-head Self-Attention (MSA), and Multiple Layer Perception (MLP) form the main components of the Transformer encoder. Therefore, the operations within FFM can be expressed as

(5) $${\dot{X}}_{l}=G_{msa}\left(G_{ln}\left(X_{l-1}\right)\right)+X_{l-1}\;l=1,2,\ldots ,L_{T}$$

(6) $$X_{l}=G_{mlp}\left(G_{ln}\left({\dot{X}}_{l}\right)\right)+{\dot{X}}_{l\;\;}l=1,2,\ldots ,L_{T}$$

Here, $G_{msa}\left(\cdot \right)$ denotes the MSA operation, $G_{mlp}\left(\cdot \right)$ denotes the MLP operation, and $G_{ln}\left(\cdot \right)$ denotes the LN operation. Additionally, $L_{T}$ represents the number of blocks. It can be observed that residual connections are employed within the Transformer encoder, effectively mitigating the vanishing gradient problem and model degradation issues. Moreover, LN plays a crucial role in Transformer models by reducing training time and enhancing generalization capabilities. The multi-head self-attention module further boosts the model’s representational power, which will be detailed below.

(7) $$Q=ZW^{Q}$$

(8) $$K=ZW^{K}$$

(9) $$V=ZW^{V}$$

Here, *Q* denotes the Query vector, *K* represents the Key vector, and *V* signifies the Value vector. Within the self-attention mechanism, the input feature *Z* must first undergo a linear transformation to yield *Q*, *K*, and *V* of identical dimensions. $W^{Q},W^{K}$, and $W^{V}$ are mapping matrices, and the dimensions of Q, K, and V are all $d_{k}$. Multiplying Q and K computes the similarity between each query vector and all key vectors, then the resulting similarity is used to calculate the weighted sum between each query vector and all value vectors. Note that the Scale function controls the numerical range of the Q × K dot product via the scaling factor $\sqrt{d_{k}}$, ensuring stability in softmax computation and gradient calculation. The attention function is expressed as

(10) $$Attention\left(Q,K,V\right)=softmax\left(\frac{{QK}^{T}}{\sqrt{d_{k}}}\right)V$$

Here, *Attention*(∙) denotes the self-attention operation.

Finally, stacking multiple self-attention mechanisms yields a multi-head self-attention mechanism. This approach mirrors the use of multiple convolutional kernels in CNNs, where learning features with different semantic meanings enhances the model’s representational capacity. The formula for multi-head self-attention can be expressed as

(11) $$\tilde{M}=\&MultiHead\left(Q,K,V\right)=\&Concat\left({head}_{1},{head}_{2},\ldots ,{head}_{h}\right)W^{O}$$

(12) $${head}_{i}=Attention\left(QW_{i}^{Q},KW_{i}^{K},VW_{i}^{V}\right)$$

Here, $W_{i}^{Q/K/V}\in R^{d_{k}*d_{k}}$, $W^{O}\in R^{hd_{k}*d_{model}}$.

## 4. Results

Each subject provided 20 sets of emotional data, comprising five instances of each emotion. For EEG, emotional features were extracted using one-second non-overlapping time windows; for facial expressions, the first frame of each video segment per second was selected for feature extraction. Each modality yielded 4407 samples. For experimental validation, five-fold cross-validation was employed. To prevent data leakage, the 20 emotion sets were divided into five folds, each containing one clip each of happy, calm, sad, and fearful expressions. The “feature concatenation” and “decision-layer fusion” used the same unimodal encoders (EFEM/FEFEM) and training protocol, so the only change is the fusion mechanism. During training, four folds served as the training set, with the remaining fold as the validation set. The final results represent the average outcome across all five-fold experiments.

### 4.1. Experimental Results of FEFEM

When selecting facial expression feature extraction networks, we compared the emotional recognition performance of six established deep learning models (LeNet, AlexNet, VGG-16, GoogLeNet, VGG-19, and ResNet) on facial expression image data from 15 hearing-impaired subjects. The model hyperparameter settings are detailed in Table 1. The optimizer employed was Adam, with a learning rate of 10^−3^ and a batch size of 32. Training proceeded for 100 iterations, utilizing the cross-entropy loss function.

During the training of deep learning models, to prevent the model from becoming trapped in local optima, we employ a cosine annealing strategy (Cosine Annealing LR) to regulate the learning rate throughout the training process. The formula is as follows:

(13) $$\eta_{t}=\eta_{min}+\frac{1}{2}\left(\eta_{max}-\eta_{min}\right)\left(1+\cos\left(\frac{T_{cur}}{T_{max}}\pi \right)\right)$$

Here, $\eta_{t}$ denotes the current learning rate, while $\eta_{min}$ and $\eta_{max}$ represent the minimum and maximum values of the learning rate, respectively. $T_{cur}$ indicates the current iteration count, and $T_{max}$ denotes the maximum iteration count. In this paper, the initial learning rate is set to 10^−3^, and the learning rate is updated once per iteration according to the cosine annealing rule.

**Figure 6:**
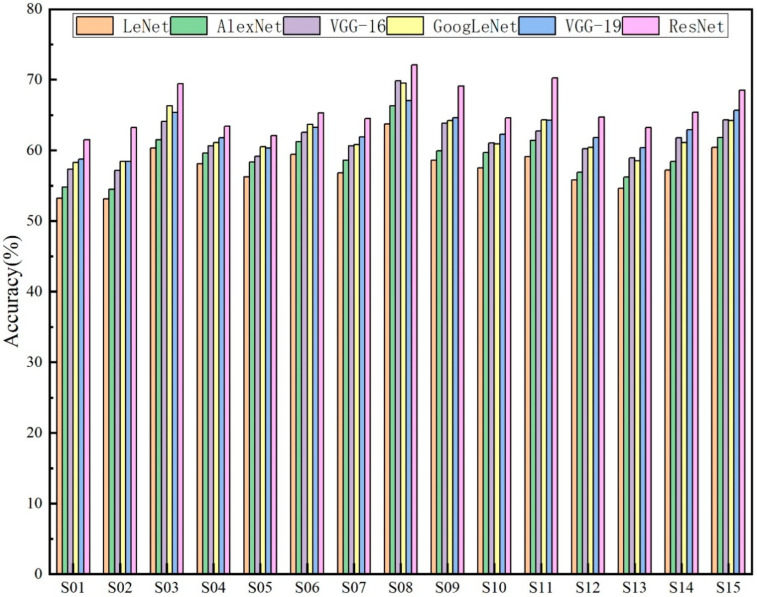
The classification accuracy of various models across different subjects.

The experimental results of FEFEM are shown in Figure 6. The average accuracy of the ResNet model reached 65.75%, the VGG-19 model achieved an average accuracy of 62.91%, the GoogLeNet model reached 62.16%, the VGG-16 model attained 61.62%, the AlexNet model recorded 59.28%, and the LeNet model achieved 57.61%. Concurrently, from a holistic perspective, across the subject-dependent experiments involving 15 participants, the ResNet model demonstrated consistently superior emotional recognition performance compared to the other five models. This further indicates that the ResNet architecture is more effective at extracting deep features associated with emotions from facial expression images.

**Figure 7:**
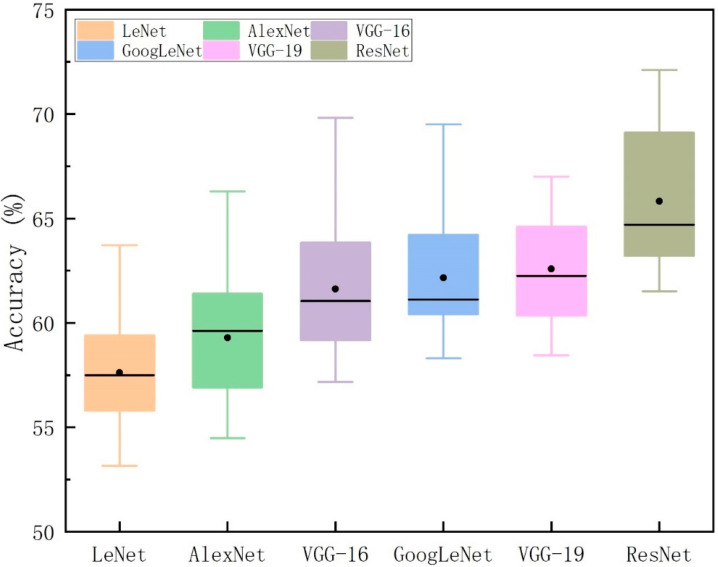
The box plots of classification accuracy across different models.

The box plot in Figure 7 illustrates the classification accuracy performance of six distinct neural network architectures. By analysing the median, interquartile range, and outliers for each model’s effectiveness, a comprehensive understanding of their accuracy performance and variability in classification tasks is achieved. ResNet demonstrated the strongest performance across all models, achieving the highest median accuracy (65.31%) and exhibiting superior consistency in performance compared to others. This indicates that its deep architecture is better equipped to capture complex emotion features. VGG-16 and VGG-19 follow closely, exhibiting high median accuracy and relatively low variability. GoogLeNet and AlexNet also demonstrate stable performance, though their accuracy slightly trails the VGG variants. In contrast, LeNet5, owing to its shallow network structure, exhibits the lowest median accuracy and greatest variability, struggling to effectively handle more complex facial expression classification tasks.

### 4.2. Experimental Results of EFEM

To evaluate the effectiveness of the proposed EFEM feature extraction method, this paper compares its emotion recognition results with those of Support Vector Machine (SVM), K-Nearest Neighbours (KNN), Gaussian Naive Bayes (GNB), Random Forest (RF), Linear Discriminant Analysis (LDA), and Adaptive Boosting (AB). DE was employed as the feature input, with other parameter settings detailed in Table 2.

The emotion recognition results of various classifiers on 15 hearing-impaired subjects are shown in Table 3. Overall, the EFEM deep learning approach demonstrated the best performance, achieving an average recognition accuracy of 66.85%, significantly higher than the other six machine learning classifiers. Compared to the other four machine learning classifiers, the SVM and LDA classifiers demonstrated relatively better performance. For several subjects, their accuracy rates also exceeded 60% (e.g., S05, S06, S11, and S12), though there were instances where accuracy fell below 30% (e.g., S03 and S08). This stems from the fact that machine learning classifiers rely on manually extracted features, which perform poorly when processing highly non-linear EEG signals.

**Figure 8:**
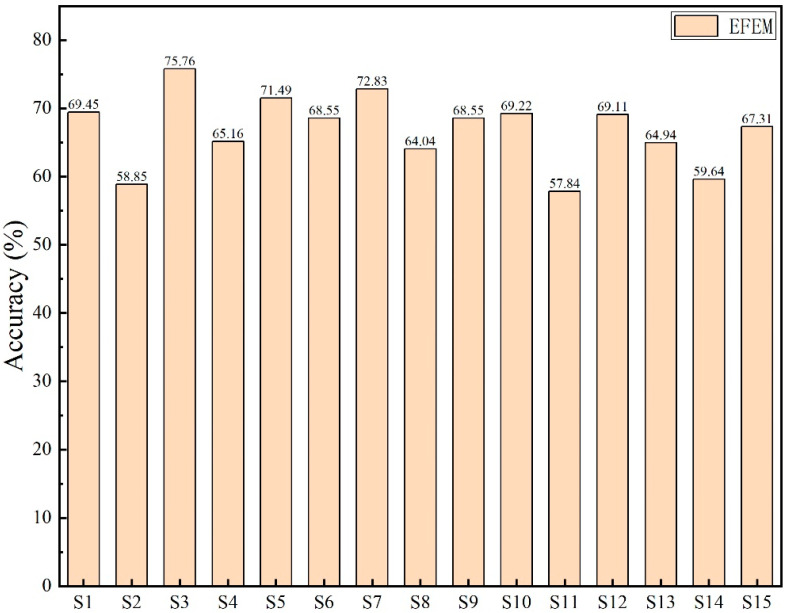
The EFEM emotion recognition results.

The standard deviation (STD) of the results further indicates that machine learning exhibits unstable performance in EEG-based emotion recognition tasks. In contrast, EFEM not only achieves a high average accuracy but also demonstrates a smaller standard deviation in its results, suggesting that deep learning methods possess stronger feature learning capabilities and generalization abilities in emotion recognition tasks. Figure 8 provides a more intuitive illustration of EFEM’s emotion recognition outcomes using single-modal EEG data. The figure reveals that subjects S02 and S11 exhibited relatively poor recognition results, achieving accuracy rates below 60%. Subject S03 demonstrated the highest emotion recognition accuracy, reaching 75.76%.

### 4.3. Experimental Results for MMHA-FNN

**Figure 9:**
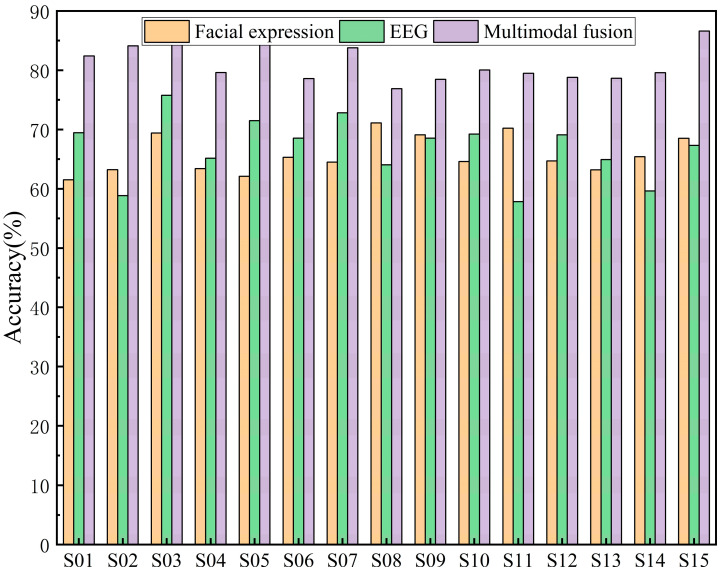
The emotion recognition results for MMHA-FNN.

The comparison of classification accuracy among 15 hearing-impaired subjects in an emotion recognition task based on different modalities (facial expressions and EEG signals) and their multimodal fusion is presented in Figure 9. The figure demonstrates that the multimodal fusion approach exhibits a significant advantage, with accuracy consistently higher than that of single-modality facial expressions or EEG alone. This indicates that multimodal fusion strategies effectively leverage the complementary nature of facial expressions and EEG signals to enhance emotion recognition performance. Overall, the EEG modality demonstrated superior emotion recognition accuracy for most subjects compared to the facial expression modality. This is because, as a physiological signal, EEG is less susceptible to masking than the non-physiological facial expression modality, thereby more accurately reflecting the subject’s genuine emotional state. However, for a minority of subjects, facial expressions demonstrated superior emotion recognition performance to EEG. This discrepancy arises because EEG signals are susceptible to noise interference. Consequently, multimodal fusion methods effectively mitigate this weakness, thereby enhancing the accuracy and robustness of emotion recognition.

As shown in Table 4, the average results of single-modal versus multi-modal emotion recognition are compared. Experimental findings indicate that multi-modal fusion achieves optimal emotion recognition performance at 81.14%, surpassing both EEG single-modal (66.85%) and facial expression single-modal (65.75%) approaches. The average accuracy of EEG single-modal emotion recognition is marginally higher than that of facial expression single-modal.

To further evaluate the trade-off between computational overhead and performance of the proposed method, this study compared the results of different models across dimensions including number of parameters (in millions), computational effort (GFLOPs), training duration (hours), and classification accuracy (%), trained on an NVIDIA RTX 3090 GPU, as shown in Table 5. It can be observed that the traditional ResNet-18 model exhibits the lowest parameter count and FLOPs, yet its accuracy rate of merely 65.7% falls short of meeting the demands of multimodal emotion recognition. Representative recent multimodal fusion approaches, such as Cross-Attention Transformer and Graph Fusion (GCN + Transformer), achieve significant accuracy improvements (79.2% and 80.5%), albeit with relatively higher parameter counts and computational complexity. We conducted a comparative analysis with reference [6]. Although both studies employed MED-HI electroencephalogram topography and facial expression data, the proposed MMHA-FNN introduced feature-level interaction fusion technology based on Transformers, thereby achieving an approximate 3% improvement in accuracy. The proposed MMHA-FNN achieves an average accuracy of 81.1% while maintaining low computational overhead, outperforming existing comparative methods.

In addition, we performed Leave-One-Subject-Out (LOSO) cross-validation to evaluate subject-independent generalization. The proposed MMHA-FNN achieved an average accuracy of 78.2%, significantly higher than decision-level fusion (67.4%) and feature concatenation (73.1%). These results confirm that MMHA-FNN can generalize to unseen subjects, further supporting its practical applicability. Concurrently, we conducted a *t*-test experiment. Results show that the improvements of MMHA-FNN over single-modality approaches (ResNet and EFEM) and even advanced baselines (Cross-Attention and GNN Fusion) are statistically significant (*p* < 0.05).

## 5. Discussion

The proposed MMHA-FNN demonstrates significant advantages in multimodal emotion recognition tasks for hearing-impaired subjects. Experimental results indicate that EEG and facial expression features exhibit distinct complementarity: EEG directly reflects neural activity during emotional processing, offering robust physiological authenticity and stability; whereas facial expressions provide overt emotional cues more closely aligned with real-world communication scenarios. Their integration enables the model to simultaneously capture intrinsic physiological responses and extrinsic expression changes, thereby substantially enhancing overall recognition performance.

**Figure 10:**
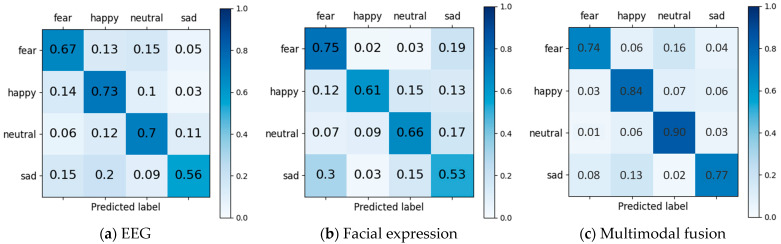
The confusion matrix for different modality experiments.

Analysis of the results indicates that multimodal fusion effectively enhances emotion recognition performance. To further investigate the complementarity between EEG and facial expression features during multimodal fusion, this study analysed the confusion matrices across different modalities for 15 hearing-impaired subjects. The results are presented in Figure 10. Figure 10a presents the confusion matrix results based solely on EEG. It is evident that EEG features demonstrate optimal performance in recognizing ‘happy’ and ‘neutral’, achieving 73% and 70% accuracy, respectively. However, recognition accuracy for ‘sad’ is notably lower, at only 56%. Figure 10b presents the single-modality facial expression-based emotion recognition results. Here, the ‘fear’ emotion achieved the highest recognition accuracy at 75% on average. Conversely, the accuracy for ‘sad’ was low at 53%, with 19% of ‘fear’ emotion samples misclassified as ‘sad’. This may stem from both emotions belonging to the negative category, which are prone to confusion in single-modal facial expression recognition. Figure 10c presents the confusion matrix for multimodal fusion-based emotion recognition. It demonstrates that the recognition accuracy for ‘sad’ improved by nearly 20% post-fusion, reaching 77%. Although the multimodal recognition accuracy for “fear” was slightly lower than that achieved through facial expression analysis alone, the overall multimodal fusion approach demonstrated greater robustness. This approach effectively leverages the complementary strengths of different modalities, thereby enhancing the overall effectiveness of emotion recognition.

To further examine how MMHA-FNN leverages cross-modal information, we quantified the average attention weights assigned to EEG frequency bands and facial regions across all subjects. As shown in Table 6, the δ and *θ* bands received the highest weights (0.28 and 0.24, respectively), indicating their strong role in capturing sadness and calmness. In contrast, β and γ bands were relatively more influential in happy and fear classification. For facial features, the eye and mouth regions contributed most (0.31 and 0.27), aligning with prior findings that emotional salience often manifests in these areas. One limitation is that we selected the first frame per second for facial streams. This may introduce sampling bias. Future work should average frames across each second or use a lightweight temporal module to better capture dynamics.

In addition to its methodological contributions, this work also provides important translational implications. The distinctive activation patterns observed in the parietal and occipital lobes of hearing-impaired subjects indicate that emotional processing in this population relies more heavily on visual and spatial pathways than auditory cues. This neurological characteristic not only supports the integration of EEG and facial modalities within the proposed MMHA-FNN framework but also motivates the development of multimodal attention mechanisms capable of adaptively weighting cross-modal information.

From a broader perspective, this study represents an early step toward practical affective interfaces for special-needs users. While the present implementation employs high-density EEG and laboratory-based recordings, future research will aim to translate these findings into portable, reduced-channel, and wearable systems that can monitor emotional states in real-world settings. Such developments could contribute to rehabilitation training, emotional feedback therapy, and assistive communication technologies for hearing-impaired individuals, ultimately bridging neuroscience insights with human–computer interaction applications.

## 6. Conclusions

This paper addresses the specific requirements for emotion recognition in hearing-impaired subjects by proposing a multi-modal fusion neural network (MMHA-FNN) based on a multi-head attention mechanism. The model extracts spatio-temporal features from EEG signals via DE brain topography, integrating these with facial expression feature representations derived from a ResNet-based architecture. At the feature layer, a Transformer multi-head self-attention mechanism facilitates dynamic cross-modal interaction and weighted fusion. Experimental results demonstrate limitations in single-modal methods’ recognition accuracy, with EEG and facial expressions achieving 66.85% and 65.75%, respectively. Multimodal fusion significantly enhances overall performance. The proposed MMHA-FNN achieves an average accuracy of 81.14% across four emotion recognition tasks in the MED-HI database, outperforming traditional concatenation fusion and decision-layer fusion approaches.

## Figures and Tables

**Table 1:** Hyperparameter tuning.

Category	Parameters
Number of iterations	100
Optimizer	Adam W
Batch_size	32
Loss function	Cross-entropy
Learning rate	10^−3^
Learning rate controller	Cosine Annealing LR

**Table 2:** Classifier parameter design.

Category	Parameters
SVM	Sigmoid Kernel C = 0.5
KNN	N_neighbors = 5
GNB	Gaussian NB
RF	N estimators = 7
LDA	N components = 2
AB	N estimators = 50 Learning Rate = 1.0

**Table 3:** The emotion recognition results for different classifiers.

	SVM	KNN	GNB	RF	LDA	AB	EFEM
S01	42.39	40.92	42.73	43.29	45.32	32.58	69.45
S02	41.26	30.89	35.74	32.47	33.03	20.18	58.85
S03	11.39	35.96	23.11	21.2	15.78	22.77	75.76
S04	52.54	42.62	46.67	37.09	55.47	43.18	65.16
S05	71.82	52.76	41.83	51.41	69.33	27.06	71.49
S06	60.65	45.55	53.33	39.35	67.64	42.73	68.55
S07	23	32.47	24.58	35.29	34.95	27.51	72.83
S08	21.2	32.69	31.57	23.68	28.75	30.44	64.04
S09	29.76	28.18	20.74	29.99	39.23	22.66	68.55
S10	52.65	32.36	23.56	31.12	60.54	43.86	69.22
S11	60.88	43.97	43.97	50.96	63.59	55.02	57.84
S12	59.53	38.67	58.85	49.27	67.76	50.28	69.11
S13	26.94	34.05	64.15	40.36	31.23	52.31	64.94
S14	32.92	33.37	43.63	30.78	36.75	32.92	59.64
S15	31.91	20.52	42.95	34.95	50.73	23.34	67.31
AVG	41.26	36.33	39.83	36.75	46.67	35.12	66.85
STD	17.14	7.67	12.81	8.89	16.25	11.39	5.15

**Table 4:** The accuracy of different modal features.

Category	Method	Recognition Accuracy (%)
Facial expression	FEFEM	65.75 (±3.11)
EEG	EFEM	66.85 (±5.15)
Multimodal fusion	MMHA-FNN	81.14 (±2.94)

**Table 5:** Comparison of model complexity and performance.

Model	Number of Parameters (M)	FLOPs (G)	Training Time (h)	Recognition Accuracy (%)
ResNet-18	11.7	1.8	2.1	65.7
Cross-Attention Transformer	18.3	3.2	3.8	79.2
Graph Fusion (GCN + Transformer)	16.1	2.7	3.5	80.5
CBAM_ResNet34 [6]	16.7	2.8	3.7	78.3
MMHA-FNN	15.2	2.5	3.2	81.1

**Table 6:** Average attention weights across EEG bands and facial regions.

EEG Band	Weight	Facial Region	Weight
δ	0.28	Eyes	0.31
*θ*	0.24	Mouth	0.27
*α*	0.18	Cheeks	0.18
*β*	0.16	Forehead	0.14
*γ*	0.14	Other	0.10

## Data Availability

The data presented in this study are available on request from the corresponding author.
